# Investigating the Antibacterial Effect of a Novel Gallic Acid-Based Green Sanitizer Formulation

**DOI:** 10.3390/foods13203322

**Published:** 2024-10-19

**Authors:** Esther W. Mwangi, Moshe Shemesh, Victor Rodov

**Affiliations:** 1 Institute of Postharvest and Food Sciences, Agricultural Research Organization, The Volcani Institute, 68 HaMaccabim Road, P.O. Box 15159, Rishon LeZion 7505101, Israel; esther.mwangi@mail.huji.ac.il (E.W.M.); moshesh@volcani.agri.gov.il (M.S.); 2 Institute of Biochemistry, Food Science and Nutrition, Faculty of Agriculture, Food and Environment, The Hebrew University of Jerusalem, Rehovot 7610001, Israel

**Keywords:** food safety, *Escherichia coli*, *Listeria innocua*, 3,4,5-Trihydroxybenzoic acid, lactic acid, hydrogen peroxide, antimicrobial formulation, synergy, ROS, viable but nonculturable (VBNC)

## Abstract

The purpose of the present study was to investigate the mechanism of action of our newly developed green sanitizer formulation comprising a natural phenolic compound, gallic acid (GA), strengthened by the Generally Recognized as Safe (GRAS) materials hydrogen peroxide (H_2_O_2_) and DL-lactic acid (LA). Combining 8 mM GA with 1 mM H_2_O_2_ resulted in an abundant generation of reactive oxygen species (ROS) and a bactericidal effect towards Gram-negative (*Escherichia coli*, *Pseudomonas syringae*, and *Pectobacterium brasiliense*) and Gram-positive (*Bacillus subtilis*) bacteria (4 to 8 log CFU mL^−1^ reduction). However, the exposure to this dual formulation (DF) caused only a modest 0.7 log CFU mL^−1^ reduction in the Gram-positive *L. innocua* population. Amending the DF with 20 mM LA to yield a triple formulation (TF) resulted in the efficient synergistic control of *L. innocua* proliferation without increasing ROS production. Despite the inability to grow on plates (>7 log CFU mL^−1^ population reduction), the TF-exposed *L. innocua* maintained high intracellular ATP pools and stable membrane integrity. The response of *L. innocua* to TF could be qualified as a “viable but nonculturable” (VBNC) phenomenon, while with the other species tested this formulation caused cell death. This research system may offer a platform for exploring the VBNC phenomenon, a critical food safety topic.

## 1. Introduction

Fresh produce often acts as a transmission vector for pathogenic bacteria, such as *Escherichia coli* O157:H7, *Salmonella enterica*, or *Listeria monocytogenes*, associated with foodborne disease outbreaks [1]. This problem is especially acute for the products consumed in a minimally processed state, such as leafy greens, whose preparation lacks a killing step of thermal or non-thermal sterilization [2]. The common sanitation practices adopted by the food industry in response to this challenge, such as washing agricultural produce with chlorinated disinfectants, contribute to food safety by reducing cross-contamination through wash water. However, these measures in their allowable doses provide only limited control of microbial contamination on the produce surface [3,4]. In addition, the disinfectants can potentially generate by-products such as trihalomethanes (THMs) that have negative impacts on human health [5,6,7] and the environment [8,9].

There has been an increasing interest in using natural phenolic compounds as green alternative antibacterial agents [10], which may possess health-beneficial properties in addition to antimicrobial activity [11,12]. Prior studies reported significant microbial elimination by active phenolic compounds [13,14,15], including gallic (3,4,5-trihydroxybenzoic) acid (GA) [16,17]. GA is naturally present in various edible plants and demonstrates a range of health-beneficial and fresh-keeping properties [18]. However, the realization of this potential demands a long incubation, typically 24 to 48 h, while a successful sanitizer should manifest a potent direct bactericidal effect within minutes. The antibacterial potency of natural compounds is often inferior to conventional synthetic biocides [19], which usually presents an application challenge. Thus, performing this approach requires improvements in antimicrobial activity to maximize the biocidal efficacy of these compounds to meet food safety needs.

The enhancement of the antimicrobial potency of active agents may be reached by finding synergistic combinations whose integral efficacy is higher than the sum of the individual ingredients’ effects [20]. It was suggested that antimicrobial synergy is achieved when the combined components have different modes of action and target sites [21,22] or, conversely, exert a complementary action on a common cellular target [23]. A combined application of natural phenolics with various antimicrobial agents has been previously reported [24,25,26]. For instance, a significant reduction in *E. coli* O157:H7 counts was achieved using a synergistic combination of GA and a Generally Recognized as Safe (GRAS) food additive, lactic acid (LA), with UV-A light, while the individual treatments and their pairwise combinations resulted in minor effects. Furthermore, this synergistic combination showed enhanced inactivation of bacteria on lettuce and spinach leaves [27]. However, practical UV-A implementation in fresh food decontamination systems may encounter technical problems.

The efficient control of bacterial growth does not guarantee the bactericidal action of a decontamination agent. Several studies have demonstrated the potential of commercial sanitizers to promote the “viable but nonculturable” (VBNC) state in bacteria [28,29,30]. VBNC is a survival strategy displayed by stress-exposed bacteria that remain viable and metabolically active but exhibit no proliferation [31,32]. The VBNC state helps bacteria escape decontamination since non-growing populations are generally less susceptible to antimicrobials than the growing ones [33]. The capacity of pathogenic bacteria (existing in a VBNC state) to resuscitate and regain virulence under favorable conditions poses an obvious public health risk [34,35,36,37]. It was demonstrated that the control of *L. monocytogenes* growth by synergistic combinations of natural antimicrobials (e.g., thymol) with mild heat was in fact a VBNC case, with the pathogen resuscitating in certain food systems [38].

Our recently developed antimicrobial formulation comprises 8 mM GA potentiated by sub-lethal doses of GRAS agents: 1 mM hydrogen peroxide (H_2_O_2_) and 20 mM LA [39]. The formulation was sufficient, within 2 min contact, to control the populations of the pathogenic bacteria *E. coli* O157:H7 and *L. monocytogenes* on baby spinach with an efficacy exceeding that of the commercial sanitizers chlorine and peroxyacetic acid, thus suggesting an alternative sanitizer for fresh produce [40]. The brief incubation followed by water rinsing and dewatering by centrifugation, as well as the relatively low ingredient concentrations, ensured a lack of effect of the formulation on the palatability and sensory properties of the produce. However, the mode of antibacterial action of this alternative sanitizer formulation has not been investigated yet. In this work, we examined the phenomenon underlying the inhibitory action of the GA-based antibacterial formulations towards Gram-negative and Gram-positive bacteria.

## 2. Materials and Methods

### 2.1. Bacterial Strains and Culture Preparation

The tested microorganisms *E. coli* ATCC 25922 and *Bacillus subtilis* NCIB 3610 were obtained from the laboratories of Prof. Shlomo Sela-Saldinger and Dr. Moshe Shemesh (Dept. of Food Science, Institute of Postharvest and Food Sciences, ARO), respectively. *L. innocua* ATCC 33090, widely used as a non-pathogenic surrogate of *L. monocytogenes* [41], was purchased from ATCC. The *Pectobacterium brasiliense* local isolate Pbr 77 from potato [42] and *Pseudomonas syringae pv. tomato* DC3000 were generously provided by Dr. Doron Teper (Dept. of Plant Pathology and Weed Research, Plant Protection Institute, ARO). The strains were preserved at −80 °C in 20% glycerol stocks (*v*/*v*) before use. The bacteria were cultivated from the frozen stock using Lysogeny broth (LB) or LB agar, both in Lennox formulation (Formedium Ltd., Norfolk, UK) for *E. coli*, *B. subtilis*, *Ps. syringae*, and *P. brasiliense,* and Brain Heart Infusion (BHI) agar and broth (HiMedia Laboratories Pvt Ltd., Thane, India) for *L. innocua*. The media were prepared according to the manufacturer’s directions. A colony from a 24 h old LB/BHI-streaked plate was inoculated into an Erlenmeyer flask containing 20 mL LB/BHI broth and activated overnight for 16 h for all bacteria apart for *B. subtilis* (6 h) at 37 ± 1 °C and with continuous shaking at 180 rpm. The overnight culture inoculum was harvested on LCEN-201 centrifuge (MRC Scientific instruments, Holon, Israel) and the pellet resuspended in 0.85% saline (*w*/*v*). The suspension was then adjusted to reach approximately 10^9^ CFU mL^−1^ with reference to optical cell density at 600 nm (OD_600_) as measured by a GENESYS 10S UV-Vis spectrophotometer (Thermo Fisher Scientific, Waltham, MA, USA).

### 2.2. Chemical Stock Preparation

GA, LA, and H_2_O_2_ were purchased from Sigma-Aldrich (Merck/Millipore Sigma, Burlington, MA, USA). The stock solutions of the chemicals were prepared by dissolving appropriate amounts of the test compounds in physiological saline. The GA solution was filter-sterilized through 0.22 µm Durapore membrane filters and stored at −20 °C until use for preparing diluted working solutions. The H_2_O_2_ and LA solutions were freshly prepared prior to the experiment.

### 2.3. Culturability Assay

The inhibitory activity of the tested compounds towards the selected bacterial species was evaluated by the plate count method [43,44]. The bacteria were treated with individual compounds and their combinations by incubating 1.5 mL bacterial suspensions (10^8^ CFU mL^−1^) in 2 mL Eppendorf tubes at 22 ± 1 °C in a Lab-Line 3521 orbital shaker (Lab-Line Instruments, Inc., Melrose Park, IL, USA) at 200 rpm for 30 min bacterial suspensions in saline without the tested compounds were used as positive controls. After the incubation, 100 µL aliquots of the samples were serially diluted, plated on appropriate media (LB or BHI agar), and incubated at 37 ± 1 °C for 24 h. Minimal inhibitory concentrations (MICs) were determined as the lowest concentrations of the tested compound that resulted in the complete absence of colonies on the plate.

The interaction of the antimicrobial formulation ingredients was evaluated by the checkerboard method based on calculating fractional inhibitory concentration indices (FICIs). The FICI values for double and triple combinations were calculated as sums of the ratios of each ingredient’s concentration in an efficient formulation to the MIC value of the same compound applied alone, as presented in Equations (1) and (2), respectively [45]. In other words, the FICI values demonstrated the possibility of achieving the antibacterial effect with lower concentrations of compounds used in combination, as compared to the same compounds applied alone.

FICI_A/B_ = MIC_A(combination)_/MIC_A(alone)_ + MIC_B(combination)_/MIC_B(alone)_ (1)

FICI_A/B/C_ = MIC_A(combination)_/MIC_A(alone)_ + MIC_B(combination)_/MIC_B(alone)_ + MIC_C(combination)_/MIC_C(alone)_ (2)

In agreement with [45], FICI values < 0.8 were interpreted as synergy, between 0.8 and 4 as indifference or additive effects, and ≥4 as antagonism.

### 2.4. Bacterial Cell Viability Assays

#### 2.4.1. ATP Quantification

Intracellular adenosine 5′-triphosphate (ATP) is considered a biomarker of microbial viability [46]. The ATP levels produced by the cells were determined using the BacTiter-Glo™ (Promega Corporation, Madison, WI, USA) kit according to the manufacturer’s protocol. This method is based on the reaction of luciferin and cellular ATP in the presence of Mg^2+^, O_2_, and luciferase enzyme to produce a glow-type signal radiated as luminescent light. The luminescent signal correlates with the amount of ATP, which in turn is proportional to the number of viable cells present in the sample. The reagent was reconstituted by transferring 10 mL of thawed buffer into a vial of the substrate and mixing to obtain a homogenous solution equilibrated at room temperature before use. To get rid of extracellular ATP, the samples were washed three times with saline. A volume of 100 µL of the bacterial suspension (10^8^ CFU mL^−1^) from each dilution was mixed and incubated with 100 µL of BacTiter-Glo reagent (1:1) in white polystyrene 96-well plates for 5 min. The luminescence values were taken using a 2300 EnSpire Multilabel Reader (PerkinElmer, Turku, Finland).

#### 2.4.2. Membrane Integrity Assay

The bacterial cell membrane integrity was examined by a LIVE/DEAD BacLight™ Bacterial Viability Kit (Molecular Probes, Invitrogen, Waltham, MA, USA) according to the manufacturer’s instructions. This assay comprises SYTO™ 9 and Propidium Iodide (PI) fluorescence dyes varying in membrane permeation and spectral characteristics. Cells with compromised membranes stain red with PI, whereas cells with an intact membrane stain green with SYTO 9 [47,48]. The 100 µL aliquots of the samples were incubated with the same amount of BacLight solution in a 96-well plate for 15 min in the dark. With excitation centered at 488 nm, the green and red fluorescence intensities were measured at emission wavelengths of 530 and 630 nm, respectively, and bandwidths of 20 nm.

#### 2.4.3. Calibration of Viability Assays

The bacterial viability assays were calibrated by analyzing artificial cellular populations comprising mixed heat-inactivated 85 °C/15 min and non-treated cells in ratios ranging from 0 to 100% inactivated cells. For each mix, the results of the viability assays presented as ATP content and as membrane integrity, and compared with the actual plate counts of the same artificial population (Figure S1). In the calibration trials, a good correlation between the viability assays and the plate counts (R^2^ = 0.96 for both assays) was reached within the cell density range of 10^6^ to 10^8^ CFU mL^−1^. Therefore, for the sake of accuracy, experiments were performed at high cellular density (10^8^ CFU mL^−1^).

#### 2.4.4. Cell Viability Analysis: Testing the Antimicrobial Formulations

The 25 mL samples containing bacterial suspensions at 10^8^ CFU mL^−1^ were treated with individual antimicrobial compounds or their combinations in 250 mL Erlenmeyer flasks. The samples were incubated at 22 ± 1 °C on an orbital shaker at 200 rpm for 30 min. In each sample, the cells were collected on a 0.2 µm vacuum filter paper (Tamar Laboratory Supplies LTD, Mevaseret Zion, Israel) and resuspended in saline followed by the viability assessment described above. The assay results were presented as the percentage of viable cells relative to positive control (non-treated cellular population). In addition, the values of the ATP/CFU ratio, suggested as an indicator of VBNC state [49,50], were calculated as the ratio of ATP content determined by the BacTiter-Glo assay (% of positive control) to culturability test results (log CFU mL^−1^).

#### 2.4.5. Cell Viability Assessment by Flow Cytometry

Bacterial staining with propidium iodide (PI) to detect membrane-compromised cells [51] was carried out according to the manufacturer’s bacteria viability manual (Molecular Probes, Invitrogen, Waltham, MA, USA). The 200 µL aliquots of filtered samples (40 µm) were incubated for 15 min with PI (final concentration, 30 mM) in black 96-well plates. Fluorescence was excited by a 488 nm laser and measured using default filters of the FL3 (PERCP-A) channel for red fluorescence at 630/20 nm optical bandpass using a flow cytometer BD Accuri™ C6 Plus (BD Life Sciences, San Jose, CA, USA). The analysis was conducted at the medium flow rate of 35 μL min^−1^, with a 16 μm core for approximately 150 μL of sample and a threshold of 10,000 events. The data were analyzed by FlowJo™ v10 Software (BD life sciences, Ashland, OR, USA). Untreated samples and heat-inactivated (85 °C, 15 min) samples in saline solution defined gating, while the unstained samples facilitated the correction of background noise and exclusion of debris.

#### 2.4.6. Bacterial Viability Assay with Propidium Monoazide qPCR (PMAxx-vqPCR)

PCR-based molecular methods such as PMAxx-vqPCR have become popular techniques for sensitive diagnosis and quantification of VBNC bacteria like Listeria [51,52]. The assay with PMAxx™ dye (Biotium, Inc., Hayward, CA, USA) was performed according to the manufacturer’s instructions with minor modification. PMAxx does not penetrate intact cells but can enter dead cells with compromised membranes, irreversibly binding to their DNA upon photolysis, hence preventing such DNA from amplification by PCR [53]. Vacuum-concentrated suspensions (0.4 mL, approximately 10^9^) of untreated, TF-treated, and heat-inactivated (85 °C, 15 min) bacterial cells were incubated with 10 µM PMAxx for 10 min to allow dye penetration. The same samples without the addition of PMAxx were prepared in parallel. The samples were photolyzed for 10 min using a PMA-Lite LED Photolysis Device (Biotium) fitted with an LED of 465–475 nm emission. After incubation, the samples were pelleted and subsequently subjected to DNA extraction. Genomic DNA was extracted using a DNeasy^®^ Powerlyzer^®^ Microbial Kit (Qiagen, Hilden, Germany) as per the manufacturer’s protocol. Extracted gDNA was eluted with 50 µL nuclease-free water and stored at −20 °C until use. Purity ratios at 260 nm (A260) versus 280 nm (A280) or 230 nm (A230) and yield were determined by a NanoDrop ND-1000 spectrophotometer (Thermo Fisher Scientific, Waltham, MA, USA). DNA templates were amplified using 16S rRNA primers (F-GCTAACTACGTGCCAGCAGC, R-GCACTCCAGTCTTCCAGTTTCCA) designed specifically for *L. innocua* with a 136 bp amplicon size. The qPCR mixture contained 10 μL of 5 μL 2X Fast SYBR™ Green Master Mix (Thermo Fisher Scientific), 0.5 μL of each primer (0.5 μM), 2 μL template DNA, and 2 μL DNase-free water. The cycling parameters were 95 °C and 20 s for activation; 40 cycles of 95 °C and 3 s for denaturing; and 60 °C and 30 s for annealing/extension in an Applied Biosysytems™ 7500 thermal cycler v2.2.2 (Thermo Fisher Scientific). Fluorescence signals above the threshold were reported as the cycle threshold (C_t_). A standard curve (Figure S2) generated by plotting the C_t_ mean of serial dilution DNA templates without PMA versus the defined ratios of culturable cells (%) evaluated the linearity range to be between 10^9^ and 10^0^. Viable counts were determined using the standard curve according to C_t_ values.

### 2.5. Estimation of ROS Production in Abiotic System

The generation of reactive oxygen species (ROS) by individual formulation ingredients and their combinations was measured in cell-free saline solutions using a fluorogenic ROS reporter, the CellROX^®^ Deep Red reagent (Life Technologies, Carlsbad, CA, USA), a dye that is non-fluorescent in a reduced state and exhibits bright fluorescence upon oxidation by ROS. Importantly, the CellROX Deep Red probe detects predominantly hydroxyl and superoxide radicals [54] but has low affinity to H_2_O_2_ [55]. Therefore, this reagent allowed the generation of radicals in the presence of exogenous H_2_O_2_ to be measured. The solutions were incubated for up to 1 h at 37 °C with 5 μM CellROX Deep Red, and the fluorescence at the Ex 488, Em 640/665 nm emission maxima was measured by a plate reader with 10 min intervals. The integral ROS production estimation was based on fluorescence enhancement calculated as added area under the curves (AAUC) of fluorescence time-courses [56] as compared to the blank (saline). Measuring the abiotic ROS production in cell-free systems allowed us to understand the level of oxidative challenge all bacteria encountered initially when exposed to the formulations, irrespective of their further defense responses.

### 2.6. Statistical Analysis

The trials were performed in triplicate. For statistical analysis, the microbiological data were transformed into a logarithmic form as decimal logarithms of the CFU and expressed as means within a t-based confidence interval at a 95% confidence level. The statistical significance of the difference between the means was determined by one-way ANOVA and compared by pairwise Tukey’s HSD test (*p* < 0.05) using JMP 15 statistical software (SAS Institute, Inc., Cary, NC, USA). Different letters (*p* < 0.05) or asterisks (* = *p* < 0.05; ** = *p* < 0.01; *** = *p* < 0.001; **** = *p* < 0.0001) represent significant differences between treatments.

## 3. Results

### 3.1. Enhancing the Antibacterial Effect of GA-Based Sanitizers Through Generating Synergistic Formulations

**Figure 1:**
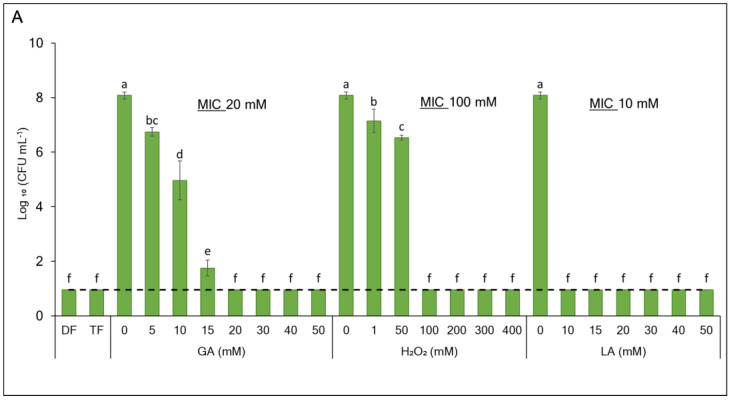

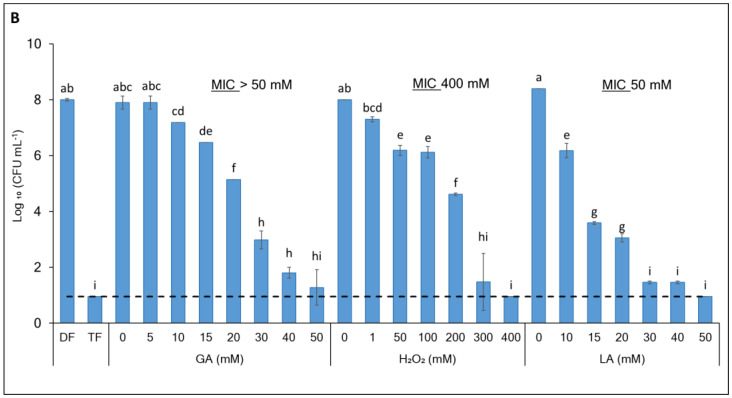
Effects of DF (8 mM GA + 1 mM H_2_O_2_) and TF (8 mM GA + 1 mM H_2_O_2_ + 20 mM LA) against (**A**) *Escherichia coli* and (**B**) *Listeria innocua* compared with different concentrations of their individual ingredients, gallic acid (GA), hydrogen peroxide (H_2_O_2_), and lactic acid (LA), enumerated as logarithmic values of colony-forming units per milliliter (Log_10_ CFU mL^−1^) on LB or BHI plates, respectively. For GA, the highest concentration tested was limited by its water solubility. (The dashed lines show the method detection limits. The values not exceeding the limit indicate lack of visible bacterial growth). Each value indicates the means of triplicate tests. Error bars represent 95% confidence intervals (*p* < 0.05). The broken horizontal line indicates the limit of detection. MIC represents the minimum inhibitory concentration. Different letters above the bars indicate significant differences (*p* < 0.05).

The synergistic antibacterial effect of the GA-based formulations in comparison to the individual formulation ingredients was comprehensively characterized for *E. coli* and *L. innocua* cells (Figure 1A,B). The dual formulation (DF) comprising 8 mM GA and 1 mM H_2_O_2_ exhibited a potent bactericidal effect towards *E. coli* (>7 log CFU mL^−1^ log reduction, *p <* 0.0001) but caused only a modest 0.7 log CFU mL^−1^ reduction (*p* = 0.856) in the *L. innocua* population. Amending the DF by supplementing 20 mM LA to yield a triple formulation (TF) resulted in the efficient eradication of *L. innocua* cells (>7 log CFU mL^−1^ population reduction, *p* < 0.0001). The comparison of the formulations’ efficacy with the MIC values of their ingredients (Figure 1A,B) revealed a substantial synergistic interaction for DF against *E. coli* (FICI 0.41) and TF against *L. innocua* (FICI 0.56), as shown in Table 1.

### 3.2. ROS Generation by Formulation Ingredients in Abiotic Systems

**Figure 2:**
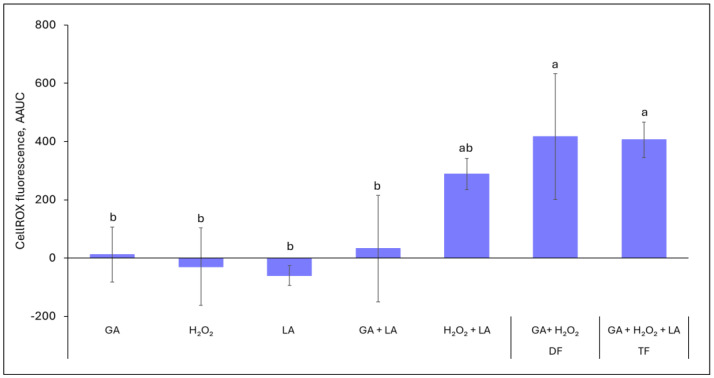
Quantification of abiotic cell-free ROS generation by individual formulation ingredients and their combinations based on CellROX Deep Red reagent fluorescence. The fluorescence intensity data were collected every 10 min over a period of 1 h and the fluorescence change was calculated as added area under the curve (AAUC) compared to the blank (saline). Each value indicates the means of triplicate tests. Error bars represent 95% confidence intervals (*p* < 0.05). Different letters above the bars indicate significant differences (*p* < 0.05).

In order to verify the involvement of ROS in the effects of the formulations, we investigated the cell-free solutions of individual ingredients and their combinations using the CellROX Deep Red reagent. Neither the individual ingredients nor the GA + LA combination demonstrated a significant enhancement of the CellROX Deep Red fluorescence (Figure 2). On the other hand, the interaction of H_2_O_2_ with LA and especially with GA (the DF) resulted in hydroxyl and/or superoxide generation, indicated by a sharp increase in fluorescence intensity. Interestingly, amending the H_2_O_2_ + GA combination with LA (the TF) did not result in additional ROS production above the level of the DF (*p* = 1).

### 3.3. Cell Viability Evaluation

**Figure 3:**
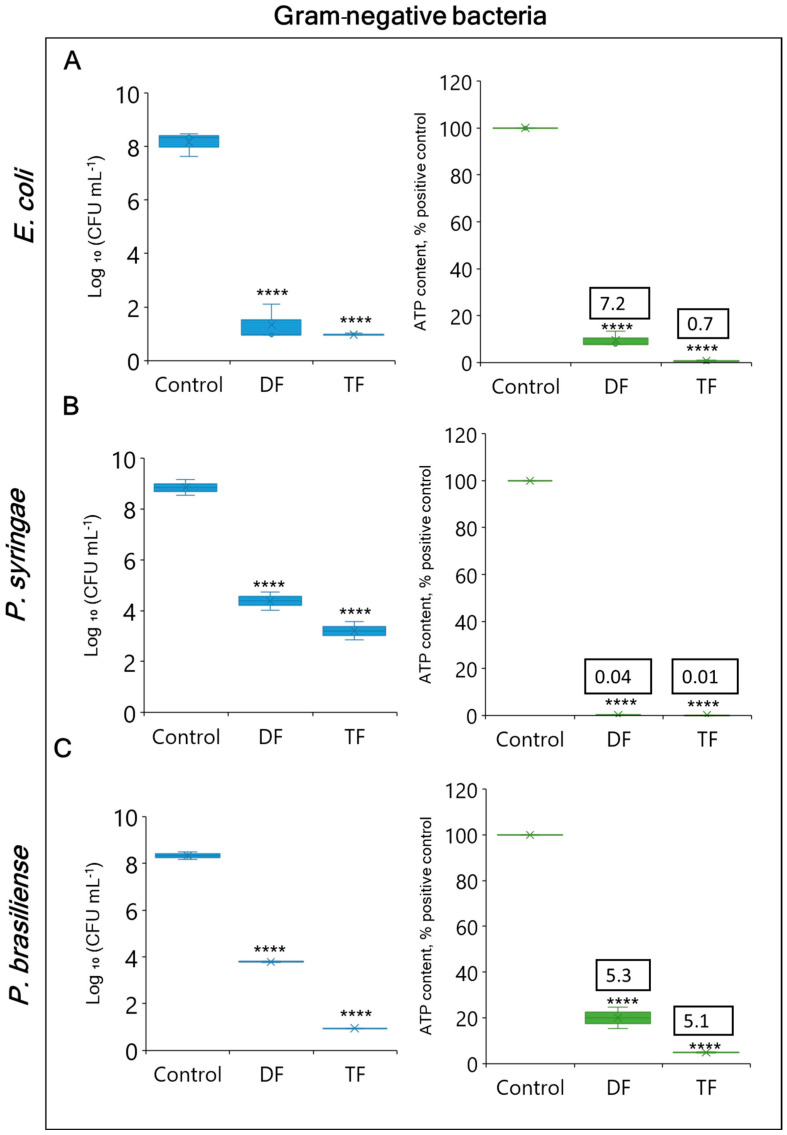
Effects of DF (GA + H_2_O_2_) and TF (GA + H_2_O_2_ + LA) formulations on viable counts of Gram-negative bacteria (*E. coli* (**A**), *Pseudomonas syringae* (**B**), and *Pectobacterium brasiliense* (**C**)) cells within 30 min incubation as determined by the plating test, with the logarithmic values of colony-forming units per milliliter (Log_10_ CFU mL^−1^) in blue box plots and the comparison to the ATP-based viability assay (ATP content % of positive control) in green box plots. Each value indicates the means of triplicate tests. The box plots represent the interquartile range of the 25th and 75th percentiles with the middle line as the median and whiskers extending to the minimum and maximum values of the data. X indicates the means of triplicate tests. Numbers in text boxes represent the ATP/CFU ratios. Asterisks indicate significant differences compared to control (*p* < 0.0001 for ****).

**Figure 4:**
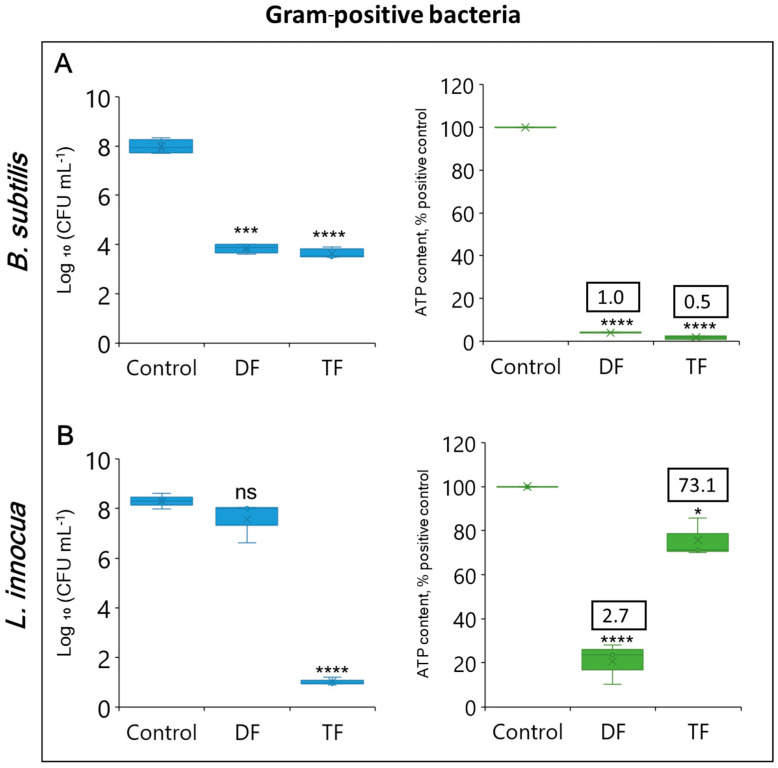
Effect of DF (GA + H_2_O_2_) and TF (GA + H_2_O_2_ + LA) formulations on viable counts of Gram-positive *B. subtilis* (**A**) and *L. innocua* (**B**) cells within 30 min incubation as determined by the plating test, with the logarithmic values of colony-forming units per milliliter (Log_10_ CFU mL^−1^) in blue box plots and the comparison to the ATP-based viability assay (ATP content % of positive control) in green box plots. Each value indicates the means of triplicate tests. Box plots represent the interquartile ranges of the 25th and 75th percentile with the middle line as the median and whiskers extending to the minimum and maximum values of the data. X indicates the means of triplicate tests. Numbers in text boxes represent the ATP/CFU ratios. Asterisks indicate significant differences compared to control (*p* < 0.05 for *, *p* < 0.001 for ***, *p* < 0.0001 for ****); ns means lack of significance.

In order to obtain an insight into the mode of action of the formulations against Gram-negative and Gram-positive bacteria, we compared their effects on bacterial culturability with ATP-based viability measurements (Figure 3 and Figure 4). Both the DF and TF had a strong inhibitory effect on the cell counts of Gram-negative bacteria, as shown in Figure 3A–C. The DF reduced plate counts by >7 log CFU mL^−1^, *p <* 0.0001 for *E. coli*, approximately 4.5 log CFU mL^−1^ for both *Ps. syringae* and *P. brasiliense* (*p <* 0.0001 and *p <* 0.0001, respectively) compared to the non-treated positive control. A complete growth inhibition of >7 log CFU mL^−1^ in *E. coli* and *P. brasiliense* (*p <* 0.0001 and *p <* 0.0001, respectively) was achieved by the TF, while *Ps. syringae* was reduced by 4.5 log CFU mL^−1^ (*p <* 0.0001). The ATP-based viability assay showed a pattern largely resembling the plating results. For the DF, the Gram-negative bacteria experienced an 80-to-100% ATP loss whereas the TF caused > 95% ATP reduction for each bacterial strain.

In Gram-positive bacteria (Figure 4A,B), the DF and the TF led to 4.1 and 4.3 log CFU mL^−1^ reductions, *p =* 0.0001 and *p <* 0.0001, respectively, for *B. subtilis*, accompanied by a considerable ATP loss of >95% (*p <* 0.0001 and *p <* 0.0001, respectively). At the same time, with *L. innocua* the DF resulted in just a minor reduction based on plate count enumeration (0.7 log CFU mL^−1^, *p <* 0.0793). A significantly greater effect on plate count of *L. innocua* (>7 log CFU mL^−1^ reduction^1^, *p <* 0.0001) was observed with the TF when the formulation was amended with 20 mM LA. The effect of DF on *L. innocua* was associated with a considerable ATP decline of approximately 80%, *p <* 0.0001. However, the addition of LA to the formulation brought about an unexpected result. In spite of the loss of culturability, the TF-treated *L. innocua* retained high intracellular ATP content (at least 70% of the untreated positive control, *p =* 0.0011), implying respiratory activity. As a result, it demonstrated a sharp increase in the ATP/CFU ratio (Figure 4B).

**Figure 5:**
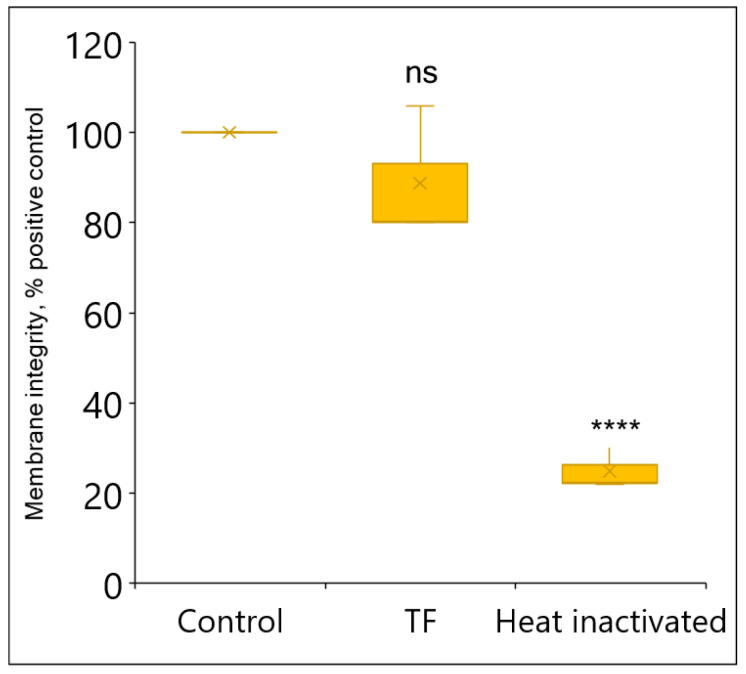
Effect of TF (GA + H_2_O_2_ + LA) on viability (% of positive control) of *L. innocua* within 30 min incubation as determined by BacLight membrane integrity assay (fluorescence units). Each value indicates the means of triplicate tests. Box plots represent the interquartile ranges of the 25th and 75th percentile with the middle line as the median and whiskers extending to the minimum and maximum values of the data. X indicates the means of triplicate tests. Asterisks indicate significant differences compared to control (*p* < 0.0001 for ****); ns means lack of significance.

**Figure 6:**
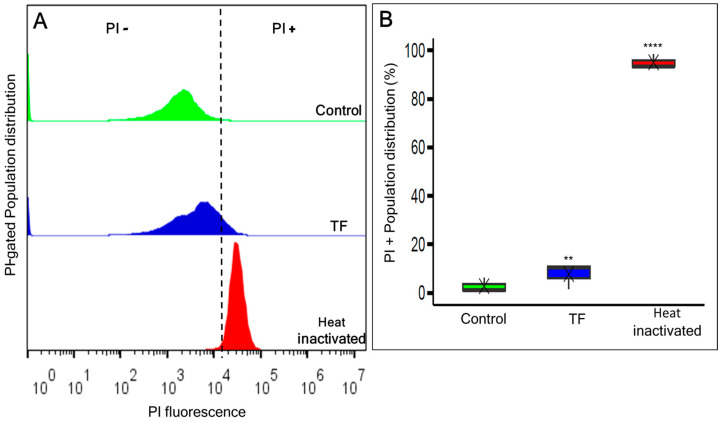
(**A**) Flow cytometry histogram profiles of *L. innocua* treated with TF (GA + H_2_O_2_ + LA) treated (blue) stained with PI shown as cell count distribution according to fluorescence intensities compared to the control (green) and heat-inactivated populations (red). (**B**) The graph summary representing the % frequency of the gated dead cells based on PI membrane extrusion. Each value indicates the means of triplicate tests. Box plots represent the interquartile ranges of the 25th and 75th percentile with the middle line as the median and whiskers extending to the minimum and maximum values of the data. X indicates the means of triplicate tests. Asterisks indicate significant differences compared to control (*p* < 0.01 for **, *p* < 0.0001 ****).

Further investigation of the TF-treated *L. innocua* cells revealed that 87% (*p* = 0.34) of them maintained membrane integrity, hence indicating cellular viability (Figure 5). Additionally, a single-cell analysis with PI staining was performed using flow cytometry (Figure 6A,B). The analysis found that 82.8% of the TF-treated cells overlapped with the control in the PI-negative zone, indicating an intact membrane. On the other hand, the heat-inactivated population displayed a dramatic increase in the membrane injury rate (PI-positive gates 98.5%, *p <* 0.0001) associated with cellular death.

**Figure 7:**
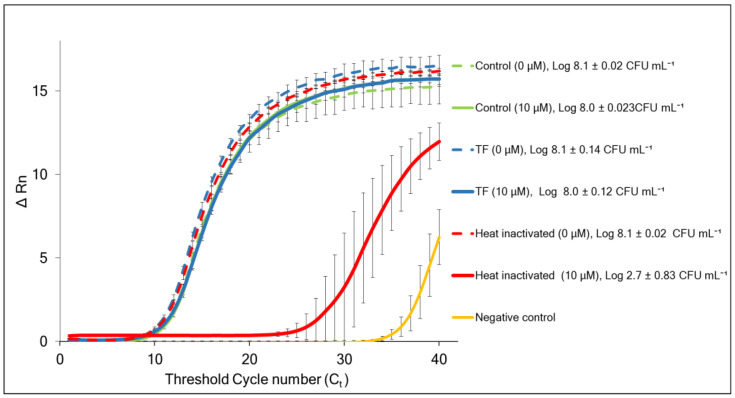
Normalized fluorescence curves of amplified DNA templates of non-treated (control), TF-treated, and heat-inactivated *L. innocua* by PMAxx-vqPCR assay targeting the 16S rRNA gene. The samples were incubated for 10 min, either with PMAxx (10 μM) or without it (0 μM), and photolysed for 15 min. Log values indicate viable cell counts derived from the standard curve ± standard deviation. Each value indicates the means of triplicate tests, the error bars represent 95% confidence intervals (*p* < 0.05), and Δ Rn represents fluorescence intensity. Negative control represents sample without DNA template.

Figure 7 shows the results of the PMAxx-vqPCR viability assay of non-treated (control), TF-treated, and heat-inactivated (dead) cells of *L. innocua*, either exposed to PMAxx (10 μM) or not exposed (0 μM). The exposure to PMAxx did not significantly affect the PCR results in the control and TF-treated cells, with all C_t_ values within a range of 11.04 to 11.72 and the calculated indicative viable cell counts between 8.0 and 8.1 log CFU mL^−1^. The lack of the PMAxx effect showed that the TF-treated *L. innocua* cells maintained membrane integrity, in spite of their growth arrest. On the other hand, PMAxx considerably reduced (*p <* 0.0001) the amplification signal of heat-inactivated cells (C_t_ = 28.17), confirming that they suffered profound membrane damage.

## 4. Discussion

This study demonstrates the antimicrobial efficacy of GA-based formulations and their potential as alternative sanitizing agents. The activity of the DF binary mix comprising GA and H_2_O_2_ was sufficient to cause significant growth inhibition of Gram-negative (*E. coli*, *Ps. syringae*, and *P. brasiliense*) and Gram-positive (*B. subtilis*) bacteria. A comparison between plate count and ATP levels revealed the bactericidal character of this inhibition, while according to the MIC values towards *E. coli*, the GA-H_2_O_2_ interaction was synergistic.

The lethality of the GA and H_2_O_2_ combination for several bacteria could be largely related to their interaction causing ROS production with subsequent cell death. The abundant generation of ROS in the GA-H_2_O_2_ mix was indicated by the CellROX Deep Red reagent. This reagent predominantly detects hydroxyl and superoxide radicals but has low sensitivity to hydrogen peroxide [54,55,57] so that it can detect radical generation in the presence of H_2_O_2_. The chemistry of the GA-H_2_O_2_ interaction in binary systems (in the absence of peroxidases, iron, etc.) has not been adequately studied and deserves further investigation. However, previous works reported that GA interaction with other oxidizing agents such as transition metals [58], blue LED light [59], UV-A [43], or UV-C [60] produced ROS, in particular hydroxyl radicals, accompanied by a potent antimicrobial effect. We suggest that similar ROS release due to GA oxidation might be a mechanistic basis of the GA-H_2_O_2_ antimicrobial synergy, in particular towards *E. coli*. Using H_2_O_2_ as an oxidizer for GA may be advantageous for applying this approach in the food industry because H_2_O_2_ has an FDA-approved status as a GRAS material [61].

It has been suggested that the generation of GA-derived ROS occurs intracellularly [62] or in the vicinity of bacterial cells because the diffusion capacity of hydroxyl radicals is limited [63]. Therefore, the antimicrobial effect of the oxidized GA might depend on the penetration of the polyphenolic substrate (e.g., GA) into bacterial cells. Gram-positive bacteria were found to be less sensitive to photo-oxidized GA than the Gram-negative ones, presumably because their cell wall structure was less permeable for phenolic compounds [63]. Indeed, in our trials, the DF mix caused a somewhat smaller population decline in Gram-positive *B. subtilis* than in the Gram-negative species, especially *E. coli*, in spite of the significant ATP loss. An alternative explanation of this discrepancy might be the presence of spores in the DF-treated *Bacillus* population. The *Bacillus* spores contain practically no measurable ATP [64], but upon germination, they may contribute to colony count in the culturability test.

A unique DF response pattern was demonstrated by another Gram-positive species, *L. innocua*. In contrast to the other species tested, the GA + H_2_O_2_ combination caused only a minor CFU reduction in *Listeria*, although its intracellular ATP pool was partially depleted, possibly for the employment of survival mechanisms [65]. The factors that alleviated the DF damage to *L. innocua* might involve low intracellular GA penetration as suggested above, ROS scavenging, or other protective mechanisms. The *Listeria* genus is known for its tolerance to a wide range of stresses, with an understandable emphasis on the highly adaptable pathogenic species *L. monocytogenes* [66,67]. *L. monocytogenes* and its non-pathogenic surrogate *L. innocua* have considerably similar stress response systems regulated by the σ^B^ transcription factor [68].

Amending the GA-H_2_O_2_ mix with a third ingredient, LA, to yield a triple formulation (TF), did not increase the ROS generation in the cell-free system. With all bacteria tested, except for *L. innocua*, LA addition just somewhat enhanced the bactericidal effect observed with DF. However, with *L. innocua*, the LA acted as a real “game changer”, switching the situation from almost uninhibited bacterial proliferation to achieving the complete prevention of *Listeria* growth when 20 mM LA was added to the mix. This remarkable activity of TF towards *L. innocua* was exhibited with ingredient concentrations well below their individual MICs, representing a case of synergistic interaction. Once TF and DF produced comparable amounts of ROS, the dramatic antibacterial effect of TF against *L. innocua* proliferation was not due to enhanced ROS generation but rather due to exerting an additional mechanism of action. One possible explanation might be facilitating the intracellular penetration of GA due to the permeabilizing effect of LA on several bacterial species, including *Listeria* [69].

LA is widely distributed in nature and extensively used in the food industry as a GRAS antimicrobial preservative [70,71], although commercial LA concentrations are 5–10 times higher than its content in TF. LA has been considered a potential alternative disinfectant for fresh produce [6,72]. However, its applications are limited due to its insufficient potency, long exposure time, and altered sensory quality [6]. While the applicability of LA as an individual sanitizer is doubtful, it may be a promising ingredient of antimicrobial formulations. Synergistic combinations of LA with H_2_O_2_ have been reported [23], and demonstrated encouraging results in decontamination trials [73,74]. Additionally, natural phenolic antimicrobials such as epigallocatechin gallate (EGCG) can sensitize *L. innocua* to the effect of LA [75].

Investigation of the effect of the TF on *L. innocua* brought about an unexpected result. In spite of the cell proliferation arrest, the treated cells surprisingly showed high intracellular ATP content, indicating the maintenance of cellular viability. Besides the ATP production, the non-growing TF-treated *L. innocua* culture preserved membrane integrity properties as demonstrated by PI staining in the plate reader, flow cytometry analysis, and molecular PMAxx-vqPCR assay. These observations were consistent with the phenomenon of the VNBC state [31]. Thus, instead of enhancing the bactericidal effect as happened with other species tested, the addition of LA to the GA-H_2_O_2_ mix shifted the *L. innocua* culture to the VBNC state. VBNC is a term used to describe an inactive state induced by stressful factors when bacteria exhibit no proliferation on nutrient media but are viable, as evidenced by detectable metabolic activity, and can undergo recovery (resuscitation) under suitable conditions [76]. VBNC entry is one of the adaptations used by *Listeria* to survive unfavorable environmental conditions such as starvation, salinity, temperature [77,78], weak acids [79], and chlorine stress [80], which are involved in the transition of *L. monocytogenes* to the VBNC state. The induction of the VBNC state in *L. innocua* has been less investigated [81,82]. To the best of our knowledge, this is the first report of the induction of VBNC in *L. innocua* by combined oxidative stress and acidity modulation.

ATP generation is a helpful indicator of metabolic activity in VBNC bacteria, including *L. monocytogenes and L. innocua* [83,84,85] as it is essential for physiological processes. In general, the VBNC state is characterized by the maintenance of a relatively high ATP level [31], and hence a sharp increase in the ATP/CFU ratio serving a VBNC marker [50], as observed in our study. At the same time, some investigations associated VBNC induction with stress-related ATP depletion [65], while resuscitation was promoted by an increase in the ATP level [86].

The mechanism of VBNC induction in our experimental system still awaits elucidation. It is plausible that the TF-treated *L. innocua* cells were under oxidative stress caused by the GA-H_2_O_2_-generated ROS. However, *Listeria* is recognized to employ a complex antioxidant system that might enable cellular survival under oxidative stress. In fact, its own generation of ROS during aerobic growth contributes to desensitization to oxidative stress in *Listeria* [87].

On the other hand, LA is not just an exogenous antimicrobial chemical but a metabolite produced by bacterial cells, including *Listeria* [88,89]. It may modulate bacterial biological functions, such as stress response, biofilm formation, or quorum sensing [72,90,91]. In *Listeria*, LA treatment induced the upregulation of numerous stress-response genes, including multiple members of the σ^B^ regulon [92]. Therefore, it is plausible that it might perform as a regulator shifting the oxidatively challenged *L. innocua* cells to the VBNC path.

To date, more than 100 bacterial species were found to be capable of entering the VBNC state, including 67 pathogenic bacteria [34]. The major food safety concern associated with the VBNC bacteria is that they can retain virulence or regain it upon resuscitation [36]. Finding ways to overcome this bacterial survival mechanism during produce decontamination is highly needed. Extending the exposure duration may result in the cellular death of the VBNC *L. monocytogenes* [79]. However, such an approach is impractical for brief sanitation treatments. Further comprehensive research is required to understand VBNC induction in *Listeria* species, in order to develop sanitation practices overcoming this bacterial defense strategy.

## 5. Conclusions

In this paper, we present for the first time the mode of antibacterial action of our innovative green sanitizer formulations comprising GA, a natural low-toxicity phenolic compound of plant origin, potentiated by GRAS food-grade compounds: H_2_O_2_ and LA. In brief, this study has brought about the following novel findings. It was discovered that the interaction of GA with H_2_O_2_, constituting the DF, resulted in abundant ROS generation and exerted a bactericidal effect (>4 log CFU mL^−1^ population reduction) on Gram-negative (*E. coli*, *Ps. syringae*, and *P. brasiliense*) and Gram-positive (*B. subtilis*) bacteria as confirmed by their intracellular ATP depletion. However, an unexpected limitation of the DF activity was encountered with Gram-positive *L. innocua* that demonstrated only a minor population reduction (0.7 log CFU mL^−1^). The addition of LA to the mix (the TF) effectively arrested the growth of *L. innocua* without changing the ROS output. The non-proliferating TF-treated *L. innocua* cells maintained cellular viability manifested as high intracellular ATP and stable membrane integrity, indicating their VBNC state. This is the first report of the induction of VBNC in *L. innocua* by combined oxidative stress and acidity modulation. This study provides a new experimental system for the further research of VBNC induction in order to overcome this bacterial adaptation strategy.

## Figures and Tables

**Table 1:** Antimicrobial effects of DF and TF on *E. coli* and *L. innocua*: interaction analysis.

Species	Formulation	GA			H_2_O_2_			LA		FICI	Interaction
		MIC_alone_	MIC_comb._		MIC_alone_	MIC_comb._		MIC_alone_	MIC_comb._		
*E. coli*	DF	20	8		100	1		10	0	0.41	Synergy
	TF	20	8		100	1		10	20	3.4	Indifferent
*L. innocua*	DF	50	n.a.		400	n.a.		50	n.a.	n.a.	n.a.
	TF	50	8		400	1		50	20	0.56	Synergy

The DF was ineffective against *L. innocua* and therefore this combination was not analyzed (n.a. = not applicable).

## Data Availability

The original contributions presented in the study are included in the article/Supplementary Materials, further inquiries can be directed to the corresponding author.

## Supplementary Materials

The following supporting information can be downloaded at https://www.mdpi.com/article/10.3390/foods13203322/s1, Figure S1: Validation of viability assays by comparison of artificial *L. innocua* cell populations of different live/dead ratios with cell viability data as detected by BacTiter Glo and LIVE/DEAD BacLight assays; Figure S2: Standard curve of qPCR assay targeting 16S rRNA genes for detection of viable *L. innocua* cells by PMAxx assay.
