# Supplementary material for: Investigating the Antibacterial Effect of a Novel Gallic Acid-Based Green Sanitizer Formulation
Source: Foods. 2024 Oct 19;13(20):3322. doi: 10.3390/foods13203322 (PMC11507653; doi:10.3390/foods13203322)
Supplement: Supplementary file 1 [file foods-13-03322-s001.zip › foods-3197362-supplementary.pdf]

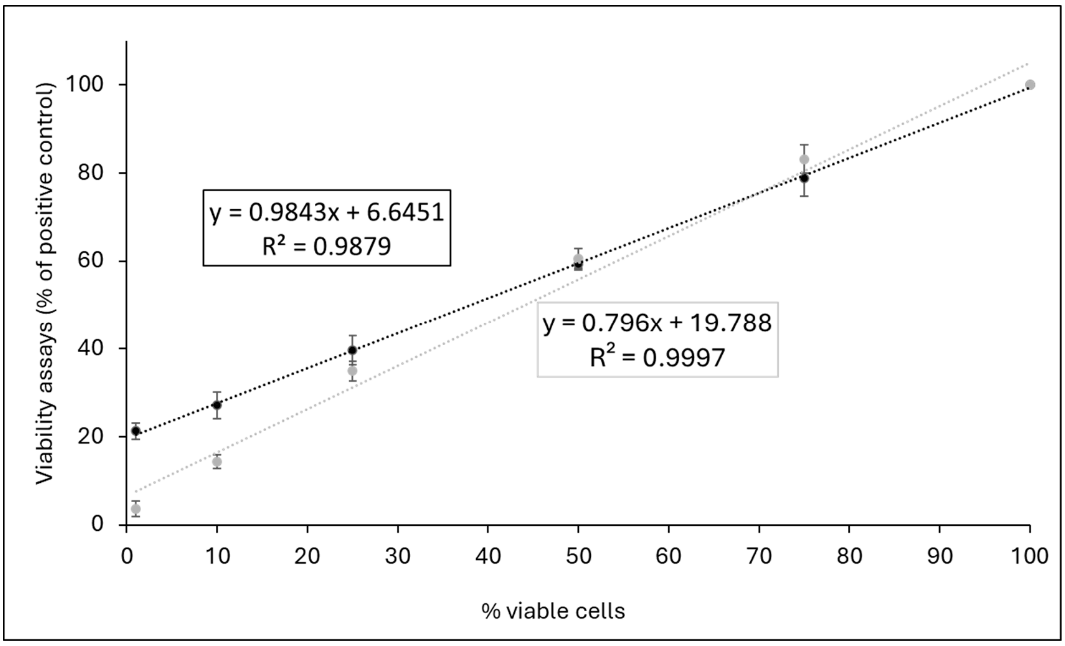

Figure S1: Validation of viability assays by comparison of artificial *L. innocua* cell populations of different live/dead ratios from 0 (10^0 CFU mL $^{-1}$) to 100% (10^8 CFU mL $^{-1}$) viable cells, versus viability data (% of positive control) as detected by BacTiter Glo (grey) and LIVE/DEAD BacLight (black) assays. Plotted points represent the mean ± confidence interval of viability values obtained from duplicate tests.

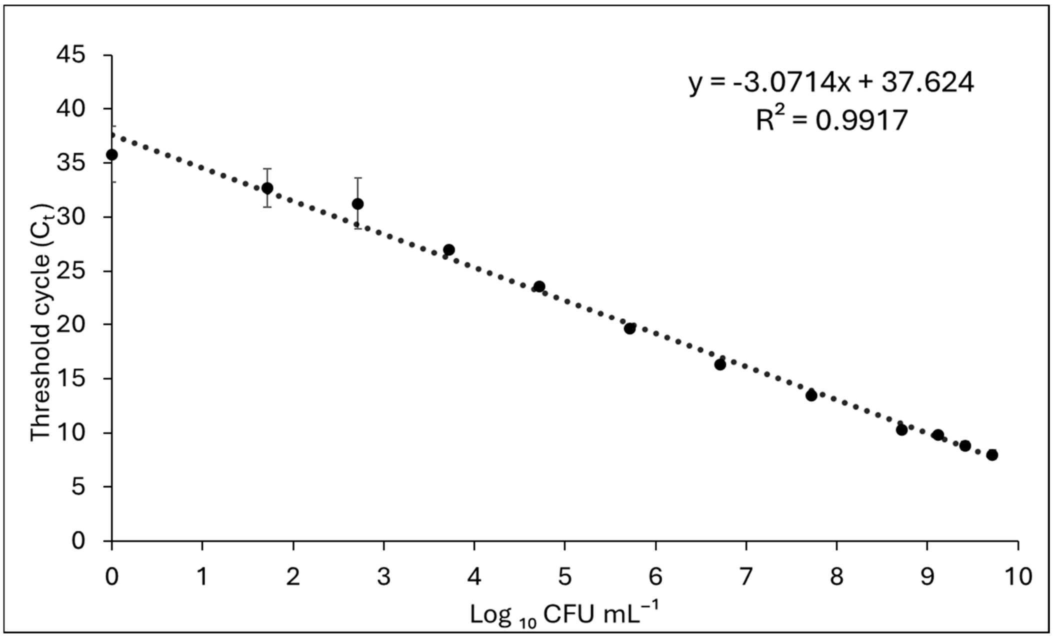

Figure S2: Standard curve of qPCR assay targeting 16S rRNA genes for detection of viable *L. innocua*. Plotted points represent the mean ± confidence interval of C_t values obtained from duplicate tests.
